# A Space-Time Adaptive Processing Method Based on Sparse Bayesian Learning for Maneuvering Airborne Radar

**DOI:** 10.3390/s22155479

**Published:** 2022-07-22

**Authors:** Shuguang Zhang, Tong Wang, Cheng Liu, Degen Wang

**Affiliations:** National Lab of Radar Signal Processing, Xidian University, Xi’an 710071, China; sg_zhang12138@163.com (S.Z.); m18392121020@163.com (C.L.); wangdg2021@163.com (D.W.)

**Keywords:** space-time adaptive processing, sparse Bayesian learning, uniform acceleration radar

## Abstract

Space-time adaptive processing (STAP) is an effective technology in clutter suppression and moving target detection for airborne radar. Because airborne radar moves at a constant acceleration, and there is a lack of independent and identically distributed (IID) training samples caused by the heterogeneous environment, using the conventional STAP methods directly cannot ensure a good performance. To eliminate these effects and improve the performance of clutter suppression, a STAP method based on a sparse Bayesian learning (SBL) framework for uniform acceleration radar is proposed here. This paper introduces the signal model of the uniform acceleration radar. To promote the sparsity, a generalized double Pareto (GDP) prior is introduced into our method, and the estimation of hyper parameters via expectation maximization (EM) is given. The effectiveness of the proposed method is demonstrated by simulations.

## 1. Introduction

Space-time adaptive processing (STAP) [1,2] is a two-dimensional adaptive filtering technique, which is widely used in the airborne early warning (AEW) radar. It plays an important role in suppressing strong clutter so that weak, slow-moving targets can be detected. Whether the STAP can achieve a good clutter suppression performance depends on the estimation accuracy of the clutter covariance matrix (CCM) of cells under test (CUT), which is usually estimated by the independent and identically distributed (IID) training samples.

Unfortunately, it is known from the Reed–Mallet–Brennan (RMB) [3] rule that the accurate estimation of the CCM requires at least twice the system degrees of freedom (DOF), and it is quite difficult to obtain in the nonhomogeneous environment. Therefore, the performance of the traditional STAP methods degrades severely.

To solve this problem, many algorithms have been proposed to ameliorate the performance of clutter suppression.

From the start of the STAP technique application, reduced-dimension and reduced-rank methods [4,5,6,7,8], including the extended factored approach (EFA) [9] and multistage wiener filter (MWF) [10], have been developed and widely used because of the relatively fewer requirements of IID training samples and faster convergence rate compared with the full STAP. The EFA method is easy to implement in practice because the DOF of radar systems can be reduced using linear transformations. However, this method still needs many training samples, especially for the systems with a large DOF. The MWF method has the advantage of a small calculation when the suitable clutter rank is selected. It is worth noting that regardless of whether the selected rank is higher or lower than the actual rank, it will greatly reduce the clutter suppression performance of the MWF algorithm. Direct data domain (DDD) algorithms [11,12] can cleverly avoid clutter inhomogeneity with just a training sample. The benefit of the DDD algorithm is achieved at the cost of aperture loss. Furthermore, KA algorithms [13,14] have been proposed, which take full advantage of parameterized models and a priori knowledge. The performance of KA algorithms relies heavily on the accuracy of a priori knowledge, and this information is hard to obtain in practice.

Over the past twenty years, the sparse recovery STAP (SR-STAP) methods [15,16,17,18] have been proposed to estimate the CCM by using the sparsity of space-time snapshot clutter data in the angle-Doppler domain, which can achieve a good performance. These kinds of methods refine the angle-Doppler two-dimensional plane into a finite grid point, and then use the information of the grid points to obtain an accurate representation of a clutter space-time profile of CUT data. The CCM is reconstructed with the obtained clutter space-time profile and the space-time dictionary matrix. The most important advantage of the SR-STAP methods is that it greatly reduces the need for training samples. However, in practical application, these SR-STAP algorithms are particularly sensitive to regularization parameters, so it is necessary to choose proper regularization parameters. Otherwise, the performance of sparse recovery will be affected, resulting in the degradation of clutter suppression.

The sparse Bayesian learning (SBL) algorithms proposed by Tipping [19] have been introduced to sparse recovery in the single measurement (SMV) case, which has drawn much attention. While retaining the advantage of sparse recovery, there is no need to choose regularization parameters. Even in the case of strong atomic correlation in the dictionary, a good performance can still be achieved. In order to utilize the information of the adjacent samples, Wipf extended it to multiple measurements (MMV) [20]. Most recently, SBL was introduced in the STAP with the MMV named M-SBL-STAP algorithm [21], which shows the potential of the SBL in the STAP.

In this paper, for the case of accelerated motion of the radar platform, the direct application of the traditional STAP method cannot achieve the equal performance of clutter suppression. Thus, we will improve the performance of the STAP method with the sparse Bayesian framework. Unlike most M-SBL-STAP methods that use Gaussian distribution priors and Laplace distribution priors, we used the generalized double Pareto (GDP) [22] prior instead in order to improve the performance of the M-SBL-STAP methods. Compared with the priors mentioned above, the GDP prior has a stronger sparsity.

The main contributions of this paper are listed as follows:

1. Based on the sparse Bayesian learning using the generalized double Pareto prior, a hierarchical Bayesian model built for the STAP is proposed here.
2. Improving the performance of clutter suppression for the radar platform that moves at a constant acceleration.
3. Compared with other STAP methods, the simulation results and performance analysis are given to demonstrate the effectiveness of the proposed method.

The work is organized as follows: Section 2 introduces the data model and Section 3 gives the details of the key theory and implements the steps of the proposed algorithm. Section 4 provides the simulation results and performance analyses of the proposed algorithm. Conclusions are drawn in Section 5.

Notations: Boldface uppercase and boldface lowercase letters are used to represent matrices and vectors, respectively; ⊗ and ⊙ denote the Kronecker product and the Hadamard product, respectively; ${\left(\cdot \right)}^{T}$, ${\left(\cdot \right)}^{*}$ and ${\left(\cdot \right)}^{H}$ denote the transposition, conjugate, and conjugate-transposition, respectively; ${\left(\cdot \right)}^{-1}$ denotes matrix inversion and $\left|\cdot \right|$ denotes the absolute value operation; ${\left\|\cdot \right\|}_{F}$ denotes the Frobenius norm and ${\left\|\cdot \right\|}_{2,0}$ stands for a mixed norm. E(·) is the expectation operation.

## 2. Signal Model and Problem Formulation

**Figure 1:**
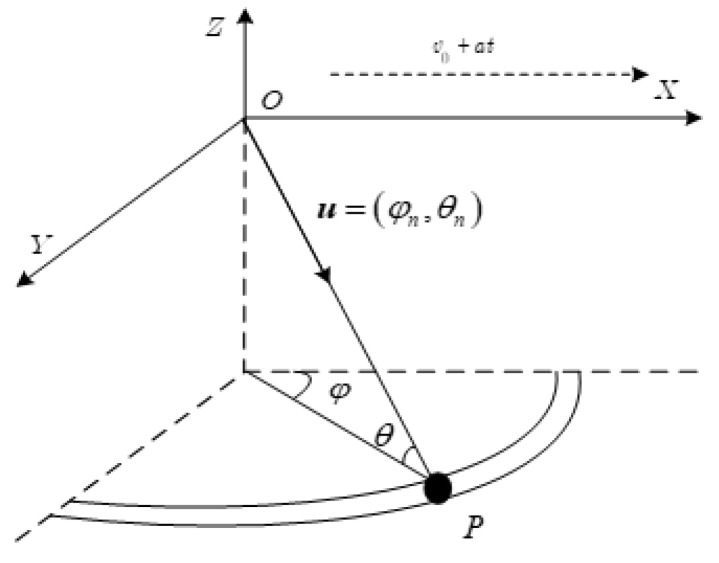
Platform geometry of airborne radar.

This paper considers the airborne radar system equipped with a uniform linear array (ULA) of N elements, and this radar system transmits M pulses at a constant pulse repetition frequency (PRF) $f_{r}$ during a coherent processing interval (CPI). The height of the radar platform is H. The azimuth and elevation angles are denoted as θ and φ, respectively. As shown in Figure 1, since this paper considers the accelerated motion of the platform and assumes that the direction of the speed and the acceleration are consistent, the airborne radar platform that moves along the X axis and its velocity and acceleration are v and a, respectively. v is often expressed as $v=(v_{0}+at)v_{d}$, where $v_{d}={\left[v_{x},v_{y},v_{z}\right]}^{T}$ (in this paper $v_{d}={\left[1,0,0\right]}^{T}$) denotes the normalized vector of the velocity direction v.

Neglecting the effect of range ambiguity, a general model for the received data of the lth range gate $x_{l}\in MN\times 1$ can be expressed as:

(1) $$\begin{array}{l} x_{l}=c+n=\sum_{n=1}^{Nc}a_{n}s_{n}^{}+n\\ =\sum_{n=1}^{Nc}a_{n}(s_{n}^{t}⊗s_{n}^{s})+n \end{array}$$

where c and n denote the clutter component and white Gaussian noise component, respectively. The Nc denotes the number of the clutter patch, which is evenly distributed in each clutter range ring. For the n−th clutter patch, $a_{n}$ is the complex amplitude and $s_{n}^{}$ is the space-time steering vector. The azimuth and elevation angles corresponding to the n−th clutter patch are $\theta_{n}$ and $\varphi_{n}$, respectively. $s_{n}^{}$ is obtained by the Kronecker product of the temporal steering vector $s_{n}^{t}$ and the spatial steering vector $s_{n}^{s}$. Due to the presence of the acceleration, it is worth noting that the temporal steering vector is different from that of a conventional uniform motion radar, but the spatial steering vector is the same. Here, we give a brief explanation. First, we assume that a scattering point P is located on the l−th range gate and its direction vector can be expressed as:

(2) $$u={\left(\cos\varphi_{n}\cos\theta_{n},\cos\varphi_{n}\sin\theta_{n},\sin\varphi_{n}\right)}^{T}$$

Then, the delay of receiving the echo from the scattering point *P* caused by the m−th pulse is given as:

(3) $$t_{P}(m)=\frac{2}{c}(\frac{v_{0}(m-1)}{f_{r}}+\frac{a{\left(m-1\right)}^{2}}{2f_{r}{}^{2}})v_{d}^{T}u$$

where c is the speed of light. We can clearly infer from Equation (3) that the Doppler frequency has changed because of acceleration. So, the $s_{n}^{t}$ and $s_{n}^{s}$ can be expressed as follows:

(4) $$\begin{array}{l} s_{n}^{t}={\left[1,\;\exp(j2\pi f_{t}),\;\cdots ,\;\exp(j2\pi (M-1)f_{t})\right]}^{T}⊙\\ {\left[1,\;\exp(j2\pi f_{t}^{\prime }),\;\cdots ,\;\exp(j2\pi {\left(M-1\right)}^{2}f_{t}^{\prime })\right]}^{T}\\ s_{n}^{s}={\left[1,\;\exp(j2\pi f_{s}),\;\cdots ,\;\exp(j2\pi (N-1)f_{s})\right]}^{T} \end{array}$$

where

(5) $$\begin{array}{l} f_{t}=2v\cos\theta_{n}\cos\varphi_{n}/(\lambda f_{r})\\ f_{t}^{\prime }=a\cos\theta_{n}\cos\varphi_{n}/(\lambda f_{r}^{2})\\ f_{s}=d\cos\theta_{n}\cos\varphi_{n}/\lambda \end{array}$$

Hence, assuming that the clutter component and the noise component are mutually uncorrelated, the clutter-plus-noise covariance matrix (CNCM) can be written as:

(6) $$\begin{array}{l} R=E(x_{l}x_{l}^{H})\\ =\sum_{n=1}^{Nc}p_{n}(s_{n}^{}){\left(s_{n}^{}\right)}^{H}+\sigma_{n}^{2}I_{NM} \end{array}$$

where $p_{n}$ denotes the clutter patch power and $\sigma_{n}^{2}$ is the noise power. $I_{NM}$ is the MN×MN identity matrix.

In order to obtain the best performance for target detection under the criterion of the maximum SINR, the optimal STAP weight vector of the lth range gate can be given by:

(7) $$w_{opt}=\frac{R^{-1}s_{t}}{{\left(s_{t}\right)}^{H}R^{-1}s_{t}}$$

where $s_{t}$ is the space-time steering vector of the target.

Combining the sparse recovery techniques with the STAP to make full use of the sparse characteristics of the clutter data, the requirements of the training samples can be significantly reduced. In the application of sparse recovery, first we discretize the angle-angular two-dimensional plane uniformly into $K=N_{s}M_{d}$ grid points, and the $N_{s}=\eta_{s}N(\eta_{s}>1)$ and $M_{d}=\eta_{d}M(\eta_{d}>1)$ represent the number of points in the spatial and temporal domains, respectively. Then, each grid point is associated with a space-time steering vector $b_{k}$, (k=1,…,K) and all of these vectors form the dictionary matrix $Β=[b_{1},\ldots ,b_{K}]\in {\mathbb{C}}^{MN\times K}$. Finally, the signal model in the STAP can be represented as

(8) X=BA+N

where $X=[x_{1},\ldots ,x_{L}]\in {\mathbb{C}}^{MN\times L}$ is the clutter data matrix consisting of L IID training samples. $A=[a_{1},\ldots ,a_{L}]\in {\mathbb{C}}^{K\times L}$ denotes the sparse coefficient matrix. Each column in A is independent and shares the same covariance matrix. $N=[n_{1},\ldots ,n_{L}]\in {\mathbb{C}}^{MN\times L}$ denotes the zero-mean Gaussian noise matrix.

According to the sparse recovery algorithms, it can be inferred that the problem of representing X is equivalent to estimating A by solving the following objective function:

(9) $$\begin{array}{l} A^{*}=\underset{A}{\arg\min}{\left\|A\right\|}_{2,0}\\ s.t.\;{\left\|X-BA\right\|}_{F}^{2}\leq \varepsilon \end{array}$$

where ε is the noise error allowance.

## 3. Proposed Method

In this section, a STAP method based on the sparse Bayesian learning (SBL) framework using GDP prior for the uniform acceleration radar is presented here.

To begin, the likelihood of measurement X has the following form:

(10) $$p(X\left|A\right.,\sigma^{2})={\left(\pi \sigma^{2}\right)}^{-MNL}\exp\left[-\sigma^{2}\sum_{l=1}^{L}{\left\|x_{l}-Ba_{l}\right\|}^{2}\right]$$

Each column in A follows a zero-mean Gaussian distribution:

(11) $$a_{l}∼CN(0,\Gamma )$$

where $\Gamma =diag({\left[\gamma_{1},\gamma_{2},\ldots ,\gamma_{K}\right]}^{T})$ and $\gamma_{k}$ is the variance for k−th row of A. 0 denotes the zero vector.

In [23], the scholars introduce the following hierarchical hyper-priors over Γ, where each hyper-parameter $\gamma_{k}$ in Γ follows the independent Gamma distribution with the new parameter $\xi_{k}$:

(12) $$p(\Gamma \left|\xi \right.)=\prod_{k=1}^{K}Gamma(\gamma_{k};\frac{3}{2},\frac{\xi_{k}^{2}}{4})$$

(13) $$Gamma(\beta ;a,b)=\Gamma {\left(a\right)}^{-1}b^{a}\beta^{a-1}e^{-b\beta }$$

(14) $$\Gamma (a)=\int_{0}^{\infty }t^{a-1}e^{-t}dt$$

Due to the introduction of the parameter, we need to model the new parameter $\xi =[\xi_{1},\cdots ,\xi_{K}]$ as independent Gamma distribution:

(15) $$p(\xi )=\prod_{k=1}^{K}p(\xi_{k})=\prod_{k=1}^{K}Gamma(h,h)$$

where h is a small constant and the value can be adjusted.

It has been demonstrated in [23] that the marginal probability distribution of $a_{l}$ follows the GDP distribution and an appropriate choice of h can promote the sparsity of the coefficient matrix A.

The prior of A can be expressed as:

(16) $$\begin{array}{l} p(A\left|\Gamma \right.)=\prod_{l=1}^{L}\prod_{k=1}^{K}CN(A_{kl}\left|0,\right.\gamma_{k})\\ \;=\prod_{l=1}^{L}CN(a_{l}\left|0\right.,\Gamma )\\ \;=\pi^{-KL}\Gamma^{-L}\exp(-\sum_{l=1}^{L}a_{l}^{H}\Gamma^{-1}a_{l}) \end{array}$$

Based on the Bayesian theorem, the posterior density of A can be obtained by combining the likelihood and prior as follows:

(17) $$\begin{array}{l} p(A\left|X,\Gamma \right.,\sigma^{2})=\frac{p(X\left|A,\sigma^{2}\right.)p(A\left|\Gamma \right.)}{\int p(X\left|A,\sigma^{2}\right.)p(A\left|\Gamma \right.)dA}\\ =\pi^{-KL}{\left|\Sigma \right|}^{-L}\exp\left[-\sum_{l=1}^{L}{\left(a_{l}-\mu_{l}\right)}^{H}{\left(\Sigma \right)}^{-1}(a_{l}-\mu_{l})\right] \end{array}$$

The posterior $p(A\left|X,\Gamma \right.,\sigma^{2})$ is a multivariate Gaussian distribution and is modulated by the hyper-parameters Γ, ξ and $\sigma^{2}$. By using the classic expectation maximization (EM) method, we can find the hyper-parameters mentioned above.

In the E-step, according to the Equation (17), covariance and mean are given as:

(18) $$\begin{array}{l} \Sigma^{new}={\left[(\sigma^{-2})B^{H}B+{\left(\Gamma \right)}^{-1}\right]}^{-1}\\ =\Gamma -\Gamma B^{H}{\left(\sigma^{-2}I_{N}+B\Gamma B^{H}\right)}^{-1}B\Gamma \end{array}$$

(19) $$\mu^{new}=(\sigma^{-2})B^{H}{\left(\sigma^{-2}I_{N}+B\Gamma B^{H}\right)}^{-1}X$$

In the M-step, we update the hyper-parameters by maximizing the expectation of J(Γ,ξ), which is defined as follows:

(20) $$J(\Gamma ,\xi )=E[\ln p(A\left|\Gamma \right.)p(\Gamma \left|\xi \right.)p(\xi )]$$

The expansion of Equation (20) is marked as Equation (21). In Formula (21), $\mu_{k}$ and $\Sigma_{kk}$ denotes the kth row of μ and the kth element on the diagonal of Σ, respectively.

(21) $$\begin{array}{c} J(\Gamma ,\xi )=E\left\{\ln\left[\begin{array}{l} \pi^{-KL}{\left|\Gamma \right|}^{-L}\exp(-\sum_{l=1}^{L}a_{l}^{H}\Gamma^{-1}a_{l})\\ \prod_{k=1}^{K}Gamma(\gamma_{k};\frac{3}{2},\frac{\xi_{k}^{2}}{4})Gamma(\xi_{k};h,h) \end{array}\right]\right\}\\ =-KL\ln\pi -L\ln\left|\Gamma \right|-\left(\sum_{k=1}^{K}\frac{\mu_{k}\mu_{k}^{H}+L\Sigma_{kk}}{\gamma_{k}}\right)+\\ \sum_{k=1}^{K}\left\{\begin{array}{l} \ln\left[\Gamma {\left(\frac{3}{2}\right)}^{-1}\right]+\ln\left[{\left(\frac{\xi_{k}^{2}}{4}\right)}^{\frac{3}{2}}\right]+\ln\sqrt{\gamma_{k}}-\frac{\xi_{k}^{2}}{4}\gamma_{k}\\ +\ln\left[\Gamma {\left(h\right)}^{-1}\right]+\ln\left[{\left(h\right)}^{h}\right]+(h-1)\ln(\xi_{k})-h\xi_{k} \end{array}\right\}\\ =constant+\sum_{k=1}^{K}\left\{\begin{array}{l} -L\ln\gamma_{k}-\frac{\mu_{k}\mu_{k}^{H}+L\Sigma_{kk}}{\gamma_{k}}+\ln\left[{\left(\frac{\xi_{k}^{2}}{4}\right)}^{\frac{3}{2}}\right]\\ +\ln\sqrt{\gamma_{k}}-\frac{\xi_{k}^{2}}{4}\gamma_{k}+(h-1)\ln(\xi_{k})-h\xi_{k} \end{array}\right\} \end{array}$$

The derivative of J(Γ,ξ) with respect to $\gamma_{k}$ is as follows:

(22) $$\frac{\partial J(\xi ,\gamma )}{\partial \gamma_{k}}=-\frac{L}{\gamma_{k}}+\frac{\mu_{k}\mu_{k}^{H}+L\Sigma_{kk}}{\gamma_{k}^{2}}-\frac{\xi_{k}^{2}}{4}-\frac{1}{2\gamma_{k}}$$

Setting Equation (22) to zero to obtain the update formula of $\gamma_{k}$:

(23) $$\gamma_{k}^{new}=\frac{1-2L+2\sqrt{{\left(L-\frac{1}{2}\right)}^{2}+\xi_{k}^{2}\left(\mu_{k}\mu_{k}^{H}+L\Sigma_{kk}\right)}}{\xi_{k}^{2}}$$

Similarly, the derivative of J(ξ,γ) with respect to $\xi_{k}$ is given as:

(24) $$\frac{\partial J(\xi ,\gamma )}{\partial \xi_{k}}=-\frac{\gamma_{k}\xi_{k}}{2}+\frac{h-1}{\xi_{k}}+\frac{3}{\xi_{k}}-h$$

Setting Equation (24) to zero to obtain the update formula of $\xi_{k}$:

(25) $$\xi_{k}^{new}=\frac{-h+\sqrt{h^{2}+2\gamma_{k}(h+2)}}{\gamma_{k}}$$

In addition to the update of the hyper-parameters, we also need to update the noise $\sigma^{2}$ at each iteration, which follows the procedure in [21]:

(26) $${\left(\sigma^{2}\right)}^{new}=\frac{\frac{1}{L}\sum_{l=1}^{L}{\left\|x_{l}-B\mu_{l}^{new}\right\|}_{2}^{2}}{NM-K+\sum_{k=1}^{K}\frac{\Sigma_{kk}^{new}}{\gamma_{k}}}$$

where $\mu_{l}$ denotes the lth column of μ. It is worth noting that $\mu_{l}$ is different from $\mu_{k}$ in Equation (21).

The M-GDP-STAP algorithm is shown in Table 1.

## 4. Performance Assessment

In this section, a simulation of the results is provided to demonstrate the effectiveness of the proposed method. Ward’s clutter model is adopted here, and the clutter-to noise ratio is set to be 40 dB. The 400th range gate is chosen to be the CUT. We set the numbers of spatial and temporal grid points to $N_{s}=4N$ and $M_{d}=4M$, respectively. The parameter h is set to be 0.1. The main parameters of a side-looking airborne radar system are listed in Table 2.

In order to evaluate the clutter suppression performance of the proposed method, we used the metric of improvement factor (IF), which is a widely used indicator to assess the performance of our proposed method and other reference methods, defined as follows:

(27) $$\mathrm{IF}=\frac{\sigma_{n}^{2}}{MN}\frac{{\left|w^{H}s\right|}^{}}{w^{H}Rw}$$

**Figure 2:**
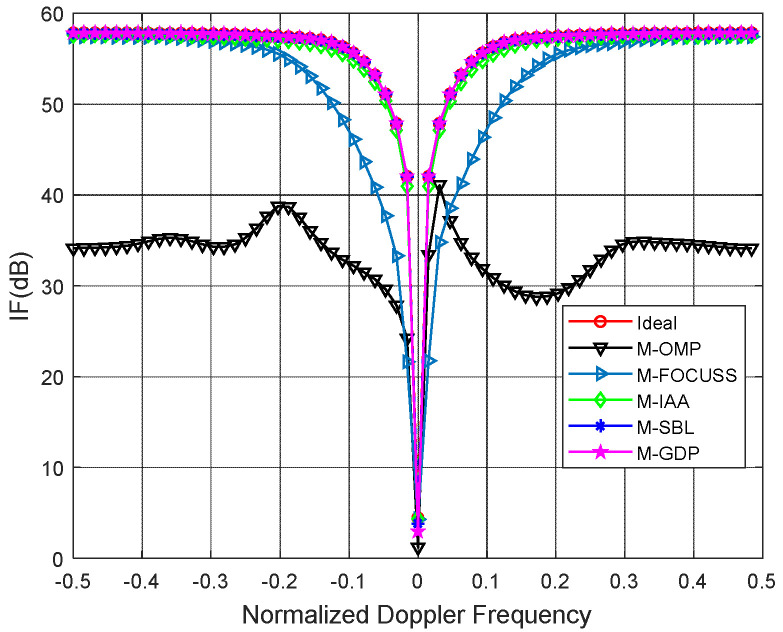
IF against normalized Doppler frequency under ideal condition.

**Figure 3:**
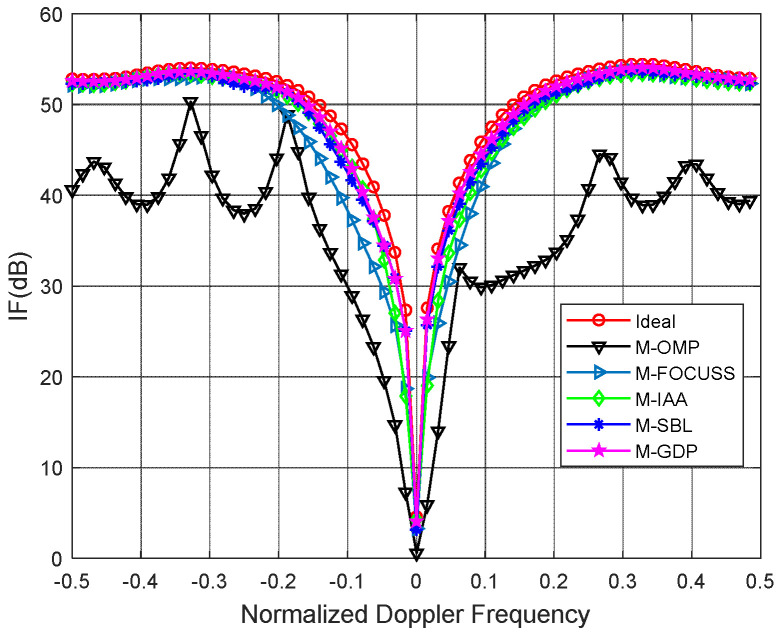
IF against normalized Doppler frequency under non-ideal condition.

The proposed method is compared with the multiple orthogonal matching pursuit (M-OMP-STAP), the multiple focal underdetermined system solver (M-FOCUSS-STAP), and the iterative adaptive approach (IAA-STAP). In addition, we also added the traditional STAP algorithm based on the angle Doppler compensation (ADC-STAP) for comparison. Figure 2 and Figure 3 plot the IF versus the normalized Doppler frequency curves under ideal and non-ideal conditions, respectively. For the non-ideal condition, we added amplitude error (standard deviation 0.03) and the phase random error (standard deviation 2) to the simulation. All simulation results of the IF are averaged in over 100 independent Monte Carlo trials.

As shown in Figure 2 and Figure 3, it can be clearly seen that the proposed method achieves better clutter suppression performance than other methods. In Figure 2, both the proposed method and the M-SBL achieve a near-optimal performance. In Figure 3, the proposed method is slightly better than the M-SBL because of the existence of amplitude and phase errors. In the simulations, the running time of the M-GDP is much less than that of the M-SBL because the latter has a slow convergence.

**Figure 4:**
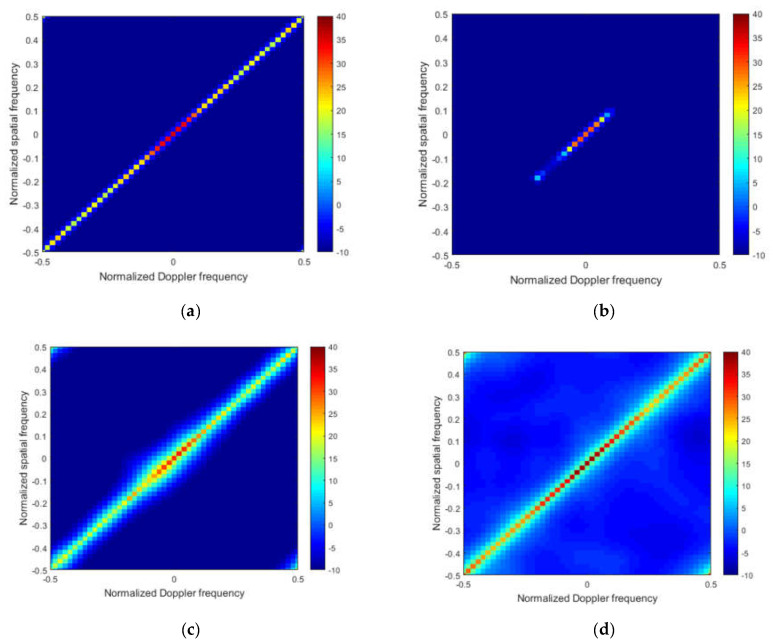

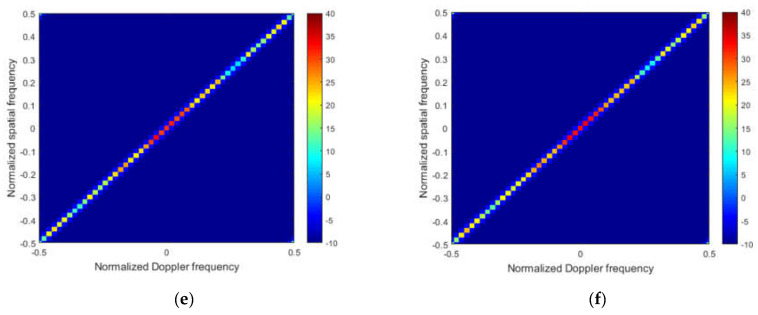
Capon spectrum of different methods without error. (**a**) The ideal capon spectrum; (**b**) the capon spectrum of M-OMP; (**c**) the capon spectrum of M-FOCUSS; (**d**) the capon spectrum of M-IAA; (**e**) the capon spectrum of M-SBL; (**f**) the capon spectrum of proposed method.

**Figure 5:**
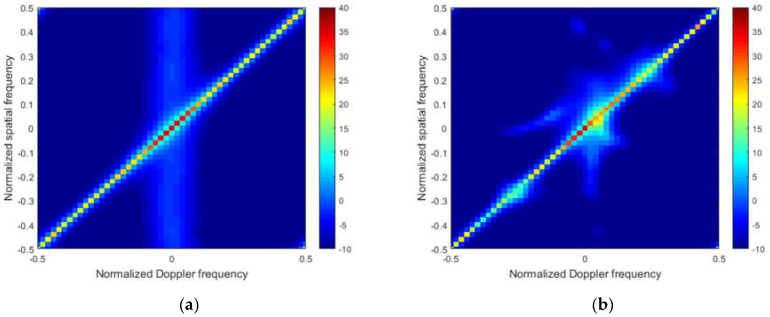

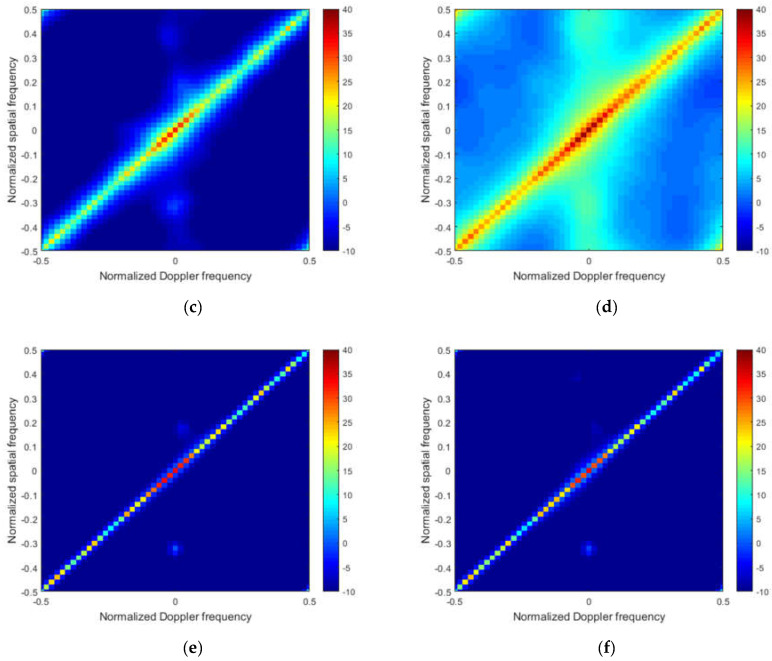
Capon spectrum of different methods with error. (**a**) the ideal capon spectrum; (**b**) the capon spectrum of M-OMP; (**c**) the capon spectrum of M-FOCUSS; (**d**) the capon spectrum of M-IAA; (**e**) the capon spectrum of M-SBL; (**f**) the capon spectrum of proposed method.

To better illustrate the performance, Figure 4a–f and Figure 5a–f plot the Capon spectra of different STAP methods under the ideal condition and non-ideal condition, respectively.

From Figure 4, in the ideal case, we note that that the clutter spectrum obtained by the M-SBL and the M-GDP are the closest to the ideal spectrum both in location and power, which indicates that the exact solution and power for clutter can both be obtained by these two algorithms. The clutter power cannot be recovered directly by the M-OMP and the M-FOCUSS from Figure 4b,c, respectively. From Figure 4d, the M-IAA overestimates the noise level, which leads to weak targets that cannot be detected.

From Figure 5, in the non-ideal case, we note that that the clutter spectrum obtained by the M-SBL and the M-GDP are the closest to the ideal spectrum both in the location and power, which indicates that the exact solution and power for clutter can both be obtained by these two algorithms. The clutter power cannot be recovered directly by the M-OMP and the M-FOCUSS from Figure 5b,c, respectively. From Figure 5d, the M-IAA overestimates the noise level, which leads to weak targets that cannot be detected.

We compared the average running time of different algorithms and the results are listed as follows: A total of 100 independent Monte Carlo trials were taken to obtain the average running time. Although the M-SBL and the M-GDP have shown a great performance in IF curves and clutter spectrums, the running time of the M-GDP is much less than that of the M-SBL. Although the running time of some algorithms is far less than that of the M-GDP, their performance cannot be accepted, such as the M-OMP, the M-FOCUSS and the M-IAA. The average running time is shown in Table 3.

## 5. Conclusions

In this paper, in order to improve the clutter suppression for the radar platform that moves at a constant acceleration, we study the model of sparse Bayesian learning and introduce the generalized double Pareto distribution prior into the SBL-based STAP framework. Compared with the classical sparse-based STAP method, the GDP prior can promote the sparsity of the sparse coefficient matrix, which will enhance the sparse recovery. At the end of this paper, the simulation results and the performance analysis are given to demonstrate the effectiveness of the proposed method.

We outline a few future research directions. For airborne radar with a higher DOF, the computational complexities of the SBL-based algorithms will be greatly improved; thus, the corresponding fast algorithm will be one of the directions the research will take. In addition, the derivation of algorithms suitable for two-dimensional array radars is also a direction worthy of research.

## Figures and Tables

**Table 1:** Pseudocode for M-GDP—STAP algorithm.

Step 1: Input the clutter data X and dictionary matrix B. Step 2: Initialize the values of the hyper-parameters ξ, γ and $\sigma^{2}$.
Step 3: E-step: Update $\Sigma^{new}$ and $\mu^{new}$ using Equations (18) and (19). Step 4: M-step: Update $\xi^{new}$, $\gamma^{new}$ and ${\left(\sigma^{2}\right)}^{new}$ using Equations (23), (25) and (26). Step 5: Repeat step 3 and step 4 until a stopping criterion is satisfied. $\frac{{\left\|\Gamma^{new}-\Gamma \right\|}_{F}}{{\left\|\Gamma \right\|}_{F}}<0.01$. Step 6: Estimate the CNCM R using the formula $R=\frac{1}{L}\sum_{l=1}^{L}\sum_{k=1}^{K}\left({\left|\mu_{l,k}\right|}^{2}\right)b_{k}b_{k}^{H}+\beta I$. where $\mu_{l,k}$ denotes the lk−th element of μ and β is the load factor. Step 7: Compute the STAP weight vector w using Equation (7).

**Table 2:** Simulation parameters of the radar system.

Parameters	Values
Number of elements in array	8
Number of pulses per CPI	8
Pulse repetition frequency Receiver bandwidth	2000 Hz 2.5 MHz
Platform height	9000 m
Wavelength Platform velocity Platform acceleration Operation frequency	0.3 m 150 m/s 50 1 Ghz

**Table 3:** The average running time.

Algorithm	The Average Running Time (s)
M-OMP	0.02
M-FOCUSS	2.63
M-IAA	1.20
M-SBL	27.83
M-GDP	3.62
